# Cost-effectiveness of train-the-trainer versus expert consultation training models for implementing interpersonal psychotherapy in college mental health settings: evidence from a national cluster randomized trial

**DOI:** 10.1186/s13012-024-01388-2

**Published:** 2024-07-29

**Authors:** Ramesh Raghavan, Ellen E. Fitzsimmons-Craft, R. Robinson Welch, Booil Jo, Enola K. Proctor, G. Terence Wilson, W. Stewart Agras, Denise E. Wilfley

**Affiliations:** 1 New York University, Silver School of Social Work, New York, NY US; 2 Washington University in St. Louis, School of Medicine, Department of Psychiatry, St. Louis, MO US; 3 Stanford University School of Medicine, Department of Psychiatry and Behavioral Sciences, Stanford, CA US; 4 Washington University in St. Louis, Brown School, St. Louis, MO US; 5 Graduate School of Applied and Professional Psychology, Rutgers, The State University of New Jersey, Piscataway, NJ US

**Keywords:** College mental health, Interpersonal psychotherapy, Cost-effectiveness analysis, Train-the-trainer, Expert consultation, Fidelity

## Abstract

**Background:**

This study is a cost-effectiveness study of two implementation strategies designed to train therapists in college and university counseling centers to deliver interpersonal psychotherapy. Costs of implementing a train-the-trainer (TTT) strategy versus an expert consultation strategy were estimated, and their relative effects upon therapist outcomes were calculated and compared.

**Methods:**

Twenty four counseling centers were recruited across the United States. These centers were randomized to either a TTT (experimental) condition, in which an in-house therapist trained other center therapists, or an expert consultation condition, in which center therapists participated in a workshop and received 12 months of ongoing supervision. The main outcome was therapist fidelity (adherence and competence) to interpersonal psychotherapy, assessed via audio recordings of therapy sessions, and analyzed using linear mixed models. Costs of each condition were quantified using time-driven activity-based costing methods, and involved a costing survey administered to center directors, follow up interviews and validation checks, and comparison of time tracking logs of trainers in the expert condition. Mean costs to produce one therapist were obtained for each condition. The costs to produce equivalent improvements in therapist-level outcomes were then compared between the two conditions.

**Results:**

Mean cost incurred by counseling centers to train one therapist using the TTT strategy was $3,407 (median = $3,077); mean cost to produce one trained therapist in the control condition was $2,055 (median = $1,932). Therapists in the TTT condition, on average, demonstrated a 0.043 higher adherence score compared to therapists in the control condition; however, this difference was not statistically significant. For the competence outcome, effect size for therapists in the TTT condition was in the large range (1.16; 95% CI: 0.85–1.46; *p* < .001), and therapists in this condition, on average, demonstrated a 0.073 higher competence score compared to those in the expert consultation condition (95% CI, 0.008–0.14;* p* = .03). Counseling centers that used the TTT model incurred $353 less in training costs to produce equivalent improvements in therapist competence.

**Conclusions:**

Despite its higher short run costs, the TTT implementation strategy produces greater increases in therapist competence when compared to expert consultation. Expanding resources to support this platform for service delivery can be an effective way to enhance the mental health care of young people seeking care in college and university counseling centers.

**Trial registration:**

ClinicalTrials.gov Identifier: NCT02079142.

**Supplementary Information:**

The online version contains supplementary material available at 10.1186/s13012-024-01388-2.

Contributions to the Literature • We present a cost-effectiveness analysis of two competing implementation strategies deployed in a national cluster randomized trial

• This is one of very few studies that have examined implementation strategy costs in relation to another implementation outcome

• This is one of the few studies to examine implementation strategy costs for interpersonal psychotherapy, an evidence-based, transdiagnostic intervention

## Introduction

Scaling up the delivery of evidence-based treatments (EBTs) in mental health requires an expansion in the capacity of providers to deliver such treatments in a high-fidelity manner. One strategy to enhance provider capacity is to train providers using a “train-the-trainer” (TTT) approach [1]. In this model, a trainer is identified (usually a therapist from within the implementation setting), who is trained in the treatment. This individual subsequently trains other therapists in the same setting; this trainer also serves as an internal coach and champion for the treatment [2]. The trainer is trained by experts, who assist in the development of the trainer both initially, as well as through longer-term consultation and supervision. The TTT model has been used for training for treatments for autism and post-traumatic stress disorder, though there exist methodological limitations in these studies (e.g., small samples, observational designs, no assessment of implementation outcomes) [3–5]. To address these limitations, we conducted a cluster randomized trial, finding superior competence in delivering interpersonal psychotherapy (IPT) in the TTT approach compared to expert-led training and consultation [2].

From an organizational perspective, one question is that of cost—an implementation outcome [6]. On one hand, inviting an expert or team of experts to train all clinicians in the EBT in one fell swoop has the virtue of simplicity. On the other hand, developing an in-house expert in the treatment, who can train several clinicians in the intervention, has the virtue of self-sufficiency and potential sustainability. How might organizational leaders make this decision as to how best to invest their resources to enhance organizational capacity to deliver EBTs? The literature seemingly provides little guidance – a recent review [7] identified only 31 studies out of 400 that examined costs as an implementation outcome, and largely during the preparation phase, not in relation to other implementation outcomes. Hence, one way to provide decision support to these decisionmakers is to compare two alternative methods of training therapists, and examine the relative impact of these two methods on implementation costs and implementation outcomes. That is the objective of this study.

As described in further detail below and elsewhere [2, 8], we evaluated the implementation of IPT delivered in college counseling centers in the United States. IPT is a highly efficacious treatment for some of the most common psychiatric disorders seen in college counseling centers, including depression and eating disorders [9–13]. College counseling centers are ideal settings in which to study the implementation of IPT because psychiatric disorders often begin in this age group [14, 15], making college students a particularly vulnerable population when it comes to their mental well-being. We compared the performance of two methods of training therapists to deliver IPT – (a) a TTT model (experimental condition) versus (b) an external expert consultation model (control condition). The main outcome of interest was therapist fidelity to IPT. In this paper, we examine the costs associated with producing fidelity outcomes in the TTT and expert consultation conditions, and compare the relative costs of achieving the same effects upon therapist fidelity under each condition.

## Methods

### Study design

Twenty four colleges with student counseling centers were cluster randomized to the two implementation strategies, matched on the ratio of the number of students per site divided by the number of therapists per site; details regarding the study, methods, and therapist outcomes are published elsewhere [2, 8]. To summarize, at colleges assigned to the experimental (i.e., TTT) condition, one therapist was selected as the trainer by the director of the study at the counseling center. This trainer attended two workshops – the first 2-day workshop trained participants in how to deliver IPT. Following this workshop, the trainer returned to their counseling center and treated up to two cases with IPT; this clinical practice was supervised, and feedback was provided to help the trainer improve. The second workshop provided the trainer with the tools to teach other clinicians how to deliver IPT, and to ensure quality control. Once the trainer had completed both workshops, they began to train their colleagues in IPT. This phase of training lasted approximately six months. Trainers met weekly with their trainees for optional group consultations and participated in optional monthly phone calls with the research team member who conducted their training and with their peer trainers from other sites.

At colleges assigned to the control (i.e., expert consultation) condition, therapists participated in a 2-day IPT workshop identical in content and structure to the first workshop delivered to therapists in the TTT condition (above). Following training, the research team member who conducted the workshop continued to engage with their trainee therapists in an optional hour-long phone call each month for up to a year in order to support them in implementing IPT.

A total of 184 professionals—95 in the TTT condition and 89 in the expert consultation condition—formed the initial cohort of therapists at participating counseling centers. Attrition of therapists occurred for reasons such as leaving the site to seek another opportunity, withdrawal of consent, retirement, and no longer seeing patients (further details regarding therapist inclusion and exclusion criteria, and the CONSORT diagram, are published elsewhere [2]. At study end, 60 therapists in the TTT condition and 55 therapists in the expert consultation condition – all of whom had audio-recorded their sessions – were included in the analysis.

### Assessing outcomes

The primary outcome measure was the change in therapist fidelity, an implementation outcome [6], which was obtained from the parent study [2, 8]. Fidelity was operationalized into two dimensions of adherence and competence, and each was assessed during two assessment timeframes (i.e., at baseline and after training). Baseline assessments were conducted before any training had occurred and captured whether therapists had prior experience with IPT. Post-training assessments were conducted after completion of all relevant workshops, within a 6–12 month window following recruitment, depending upon condition. These two dimensions were assessed from audio recordings of therapy sessions, to which therapists had consented, and of which they were aware. Assessments were conducted by raters who were masked to the implementation strategy and used the IPT Fidelity Rating Scale to determine fidelity [16]. Adherence scores ranged from 0 to 1. Competence scores ranged from 0 to 2. Raters included a senior study team member and five graduate student research assistants who were masked to implementation condition. Interrater reliability, as calculated from a sample of 9 audio recordings after training, was reported as 0.72 (95% CI, 0.46–0.91) [2].

We obtained information on changes in fidelity scores directly from the parent study [2, 8]. The parent study reported outcomes from standard linear mixed effects models conducted in Mplus version 8 [17]. Therapist outcomes were based on intent-to-treat; outcomes from all randomized therapists were included as long as a therapist had at least one post-training assessment. Models were specified as random intercept models, all standard errors were estimated using robust maximum likelihood, and effect sizes for adherence and competence outcomes were reported as Cohen’s *d*. Changes in adherence and competence were reported as *b*, which is a nonstandardized coefficient obtained from regressing the slope on centered training condition, centered baseline covariates, and their interactions. All outcomes were compared both within and across conditions.

### Costing procedures

We gathered costs of the two implementation strategies using three methods, as described below. First, we developed and fielded an implementation cost survey, which was administered before implementation activities came to an end at the site. Second, we conducted telephone follow up interviews at sites to validate and clarify survey responses. Finally, we obtained time tracking logs maintained by the research team that trained therapists across both conditions.

We used the Survey Monkey platform (www.surveymonkey.com) to deploy the implementation cost survey designed to capture labor and nonlabor costs associated with implementation. (Supplement 1 lists the key labor and nonlabor cost items gathered in the TTT condition; cost items for the control condition are a subset of those in the TTT condition.) The survey was disseminated prior to the end of all study activities at the site, was completed by the study director at each site, and was based on an time-driven activity-based costing approach [18, 19]. Items elicited details about the staff engaged in the study, salaries for therapists, amounts of time spent by therapists in each part of the implementation process, and nonlabor costs such as facility and equipment costs.

Prior pilot work on costing had revealed that many mental health clinician/administrator respondents were unused to thinking about their work in terms of costs. Hence, we conducted an hour-long, semi-structured, follow-up interview with the study director at the site, scheduled as soon as feasible following receipt of the cost survey. This interview went over the individual items listed in Supplement 1, ensuring that respondents had understood the questions, clarifying any items that were unclear, and reconfirming responses. Directors reviewed the survey items they had previously completed, consulted additional documents (if needed), and were asked to procure any other information, in order to enhance the validity of their responses. All directors participated in the interviews. Two members of the study team jointly conducted the first 4 interviews to enhance reliability.

Third, we independently attempted to increase the validity of therapist self-report using tracking logs maintained by the research team. Tracking logs are contact logs or time logs that capture the amount of time spent in specified activities. The expert maintained a tracking log of his time as he undertook training activities across both conditions. In the expert consultation (control) condition, therapists self-reported the time that they spent consulting with the expert. This self-reported time was compared with the time log of the expert who provided such consultation. In the TTT condition, trainers self-reported their time spent consulting with the expert, which was compared with the expert’s time log. However, in this condition, the training of individual therapists is overseen by the trainer within the site, not by the expert, and so we have no way to validate the time spent by the trainer in training therapists. Because we were able to validate only part of the overall labor costs in the TTT condition, we opted to rely on self-reported times, available across both conditions, in this study.

### Cost estimation

We obtained labor and nonlabor costs associated with implementing IPT. Labor costs are generated by trainers, therapists, research staff, and administrators located in participating counseling centers, as a result of performing activities associated with implementation; examples include the cost of practitioner time spent in training, and the time costs of trainers engaged in supervision and training in the TTT condition. These costs, listed in Supplement 1, were aggregated at the level of a counseling center. Nonlabor costs were direct monetary costs to centers of implementing the two conditions, and include the cost of materials and manuals, travel, and supplies. Clinicians at college counseling centers were not required to meet defined productivity metrics in terms of hours spent seeing clients. Counseling centers operated on university-established budgets and did not directly bill students or insurers for services. Our perspective is solely that of the counseling center, not of the parent university or other payors.

All 24 sites submitted surveys and participated in interviews. In the expert consultation condition, we re-interviewed 3 therapists who had reported unusually high in-house consultation hours and corrected the numbers. Missing time values of all group activities (attending expert consultation, time spent in in-house peer consultation) were filled based on mean self-reported times of other clinicians attending the same groups at the same time at the same site (i.e., group mean substitution). For individual activities (time spent in reading materials before and after training), we used mean self-reported times from all therapists in that condition (i.e., site-level mean substitution).

We entered data from the survey into a worksheet detailing time spent by individual therapists, clustered by site, on all implementation activities in order to first produce a per-therapist cost of implementation. Because of confidentiality concerns with individual salaries, time spent by therapists in implementation activities was multiplied by the site average for salary and fringe to arrive at a per therapist cost. Therapist costs were aggregated at the center level, to which we added all additional costs incurred by the center for training activities (photocopying, telephone charges, etc.). Labor and nonlabor costs of the expert were also charged to the center and added. Here, we report costs per study therapist trained, averaged across all centers assigned to each condition.

### Cost-effectiveness analysis

We calculated the difference in the mean implementation costs of TTT versus expert consultation incurred by a counseling center without discounting, given the time horizon of the study. We then divide that by (a) the difference in mean adherence scores, and (b) the difference in mean competence outcomes of therapists within that center for each implementation strategy in two separate calculations, one for each outcome. We did not examine clinical outcomes of the students (clients) served by therapists at these counseling centers.

Quantifying Thresholds for Therapist Training Outcomes.

We were unsuccessful in arriving at an a priori willingness to pay threshold to inform the cost-effectiveness analyses. Consequently, we followed the suggestions of Briggs and colleagues [20], and present uncertainty around our estimates. We estimated the joint density of variations in the incremental costs of training therapists incurred by counseling centers (i.e., difference in the mean per-therapist training cost between the conditions) and incremental fidelity outcomes (difference in outcomes between conditions) for statistically significant relationships. We ran 1000 replications of a non-parametric bootstrap re-sampling of costs (from the current study) and outcomes (from the parent trial), to populate a scatter plot.

### Sensitivity Analyses

We conducted a total of 5 sensitivity analyses – (i) As described below, counseling centers in our study trained both therapists that participated in the study (“study therapists”), as well as those that were not part of the study (“non-study therapists”). In order to quantify economies of scale among sites that trained a large number of non-study therapists, we quantified the mean per-therapist cost of training study therapists only, and the mean per-therapist cost of training all therapists regardless of participation in the study, for each center that trained non-study therapists. We then differenced these to obtain cost savings on a per-therapist basis for the sites that also trained non-study therapists. (ii) In order to test if it is more cost-effective to train highly skilled therapists as trainers, we used a design feature of the parent study, in which trainers in the TTT condition received training and supervision until they were deemed ready by the expert to train others in IPT. We calculated mean therapist costs for variations in training intensity for trainers – at the 10th percentile of post-training consultation and supervision time (suggesting that these trainers were highly skilled and did not need a great deal of training) versus the 90th percentile of training time. (iii) As we describe below, between a quarter and a third of therapists across various sites possessed prior experience in the use of IPT [2]. We calculated mean per-therapist costs for those therapists whose baseline adherence and competence scores were above the mean baseline score for all therapists in those respective conditions (indicating that these were therapists skilled in IPT even prior to training). We compared these training costs to the costs of training therapists whose baseline adherence and competence scores were below the mean. (iv) We quantified the cost implications of using therapist self-report versus expert-report when it came to quantifying training time. We did this for the expert consultation condition alone because the time taken for each task in this condition is independently quantified by both therapists and the expert. (v) We conducted sensitivity analysis for missingness in the time taken for the performance of individual tasks by therapists in the TTT condition. We used the 10th and 90th percentiles of self-reported times as our range of plausible values, and then estimated costs at these two values for all therapists in order to obtain a range.

## Results

### Counseling center and therapist characteristics

The 24 counseling centers were located in private (*n* = 7) and public institutions, of sizes ranging from 2,500 students to 51,000 students. Colleges in the experimental TTT condition were smaller (mean of 13,960 students/site) than those in the expert condition (mean of 23,475 students/site). However, the ratio of students per therapist was comparable across conditions (TTT: mean of 1,821 students/therapist; expert: mean of 1,840 students/therapist), by design. The number of therapists per site ranged from 2 to 9 (TTT condition) and 2 to 11 (expert consultation condition).

Executive directors of counseling centers were predominantly of white race/ethnicity (TTT: 83%; expert: 75%), and possessed PhDs (TTT: 83%; expert: 100%). Directors in the TTT condition were, on average, older than those in the expert consultation condition (53.3 years vs 45.6 years, respectively). Of a total of 184 therapists recruited, 95 were in the TTT condition. Therapists were largely female-identified (TTT: 81%; expert 71%), and of white race/ethnicity (TTT: 80%; expert: 74%). Mean ages of therapists in the TTT condition (42.8 years) and in the expert consultation condition (41.1 years) were comparable. Over half of all therapists possessed doctoral degrees (TTT: 56%; expert: 64%), while the rest had Masters and other professional qualifications. Therapists in the TTT condition had more years of experience in the present position (mean experience of 6.2 years; range of less than 1 year to 31 years) than those in the expert condition (mean of 4.8 years; range of less than 1 year to 20 years). None of these differences were statistically significant between conditions. Around 23% of therapists self-reported having participated in IPT-related training (workshop or class) prior to the study, and 38% reported having used IPT in their clinical work in the prior year [2].

### Implementation costs

Table 1 shows costs incurred by 12 counseling centers to implement IPT in the TTT condition by activity and by site. The mean training and supervision cost to train one trainer was $8,194 (range: $7,042 to $10,078). These trainers then trained between 2 and 9 (mean: 5.1) participating study therapists at their sites in IPT. The mean overall costs to produce one trainer and train several therapists were between $13,818 and $24,708. Overall costs to produce one therapist ranged between $2,148 and $7,117 (mean = $3,407; median = $3,077).

**Table 1:** Implementation costs of the train-the-trainer implementation strategy

Agency	Costs of training the trainer ($)			Costs of the trainer training other therapists at the center ($)		Costs to train therapists ($)	Number of therapists trained by trainer	Training costs per therapist (mean $)
	Workshop 1 training costs	Post-Workshop 1 costs	Workshop 2 costs	Site workshop costs	Post-site workshop costs			
1	2420	1612	2646	2618	4937	14,234	2	7117
2	2976	1524	2977	3757	3292	14,526	3	4842
3	2977	1112	2767	4478	3349	14,683	6	2447
4	2580	1787	2798	5688	6491	19,344	6	3224
5	2445	1898	2438	3445	1665	11,892	4	2973
6	2826	1912	2696	6151	7784	21,370	6	3562
7	3258	2130	2861	5782	4843	18,754	6	3126
8	3214	2908	3093	5923	8511	23,649	5	4730
9	2800	2200	2822	8197	8690	24,708	6	4118
10	2892	1293	2697	7537	4912	19,331	9	2148
11	2343	2201	2005	4144	3125	13,818	6	2303
12	2985	2876	2791	3394	2889	14,935	3	4978

Please see Supplement 1 for details of activities that went into these costs. Training costs are a sum of labor and nonlabor costs expended by a counseling center

As expected, the expert condition was associated with lower costs of implementation (Table 2). Sites trained between 2 and 11 therapists (mean: 5.3). Mean per-therapist training cost for all activities associated with workshop 1 (training therapists in IPT) was $1,143, and mean cost for all post-workshop consultation and supervision activities was $913. The mean overall costs to train their therapists ranged between $5,425 and $21,963. Each site spent between $1,335 and $3,661 to produce their one trained therapist (mean = $2,055; median = $1,932).

**Table 2:** Implementation costs of the expert consultation implementation strategy

Agency	Costs of training therapists ($)		Costs to train therapists ($, totals)	Number of therapists trained	Training costs per therapist ($)
	Workshop 1 training costs	Post-Workshop 1 costs			
1	6291	4823	11,114	8	1389
2	5463	2153	7616	3	2539
3	8583	13,380	21,963	6	3661
4	3596	2876	6472	2	3236
5	5159	2406	7565	4	1891
6	3400	2025	5425	2	2712
7	5956	4277	10,233	4	2558
8	4677	2641	7318	3	2439
9	8985	5698	14,683	11	1335
10	7336	6701	14,037	7	2005
11	6306	6760	13,066	7	1867
12	6241	3760	10,001	6	1667

Activities generating these costs are a subset of those shown in Supplement 1. Training costs are a sum of labor and nonlabor costs expended by a counseling centers

On a per-therapist basis, it cost a college counseling center a mean of ($3,407—$2,055 =) $1,352 more, or 66% more, to train a therapist using the TTT strategy versus an expert consultation strategy.

### Therapist outcomes

Please see Table 3 for mean scores for adherence and competence in delivering IPT before and after training. As detailed elsewhere [2], therapists in both conditions showed significant improvements over time in both adherence and competence outcomes within their group. For the *adherence* outcome, therapists in the TTT condition displayed an increase of 0.23 [95% CI: 0.19–0.27] and those in the expert consultation group displayed an increase of 0.19 [95% CI: 0.15–0.24; both *p* < 0.001] following training. Effect sizes were in the large range (TTT group: 1.64 [95% CI, 1.35–1.93]; expert group: 1.34 [1.02–1.66]). Between group differences, however were not statistically significant. Improvements in therapist scores on adherence did not significantly differ based on the condition to which they were assigned. Absolute between-group differences were 0.043 (effect size = 0.3; *p* = 0.15).

**Table 3:** Longitudinal Changes in Therapist Level Outcomes

Outcome:	Condition	Within-group differences		Between-group difference	95% CI	P value
		**Mean baseline score (range)**	**Mean post-training score (range)**			
Adherence	TTT	0.03 (0–0.25)	0.27 (0–0.6)	0.043	-0.02–0.10	0.15
	Expert	0.05 (0–0.25)	0.23 (0.04–0.7)			
Competence	TTT	0.02 (0–0.2)	0.21 (0–0.6)	0.073	0.01–0.14	0.03
	Expert	0.04 (0–0.3)	0.13 (0–0.7)			

All outcomes are measured on a 0–2 scale

Therapists in both groups also showed significant improvements within group in interpersonal psychotherapy *competence* (TTT group: 0.18 [95%CI,0.13–0.23]; expert group: 0.11 [0.06–0.15]; both *p* < 0.001). Effect size for the TTT condition was in the large range (1.16 [95% CI,0.85–1.46]; *p* < 0.001), but was medium for the expert consultation condition (0.69 [95% CI, 0.38–0.99]; *p* < 0.001). In contrast to the prior outcome, there was a statistically significant difference between groups – therapists in the TTT condition had 0.073 higher scores on competence compared to those in the expert consultation condition (95% CI, 0.008–0.14;* p* = 0.03; effect size 0.5).

### Costing analysis

Regarding the adherence outcome, centers in the TTT condition observed a mean improvement of 0.23 in therapist scores after training, while those in the control condition saw a smaller improvement of 0.19. However, between-condition differences in adherence were not statistically significant. Each 1-unit improvement in therapist adherence scores would require an investment of $14,813 under using a TTT strategy, and an investment of $10,816 using the expert consultation strategy. For the adherence outcome, the expert consultation strategy dominates. It is cheaper to train a therapist using this strategy, and there are no statistically significant differences in improvement on adherence scores between the two approaches.

Regarding the competence outcome, centers in the TTT condition observed a mean improvement of 0.179 in therapist scores after training, while those in the control condition saw a far smaller improvement in mean competence scores of 0.106. Each 1 unit improvement in therapist competence scores would require an investment of $19,033 under using a TTT strategy, and an investment of $19,386 using the expert consultation strategy – a mean saving of $353 per therapist for a counseling center adopting a TTT implementation strategy.

### Quantifying uncertainty around cost savings

**Fig. 1:**
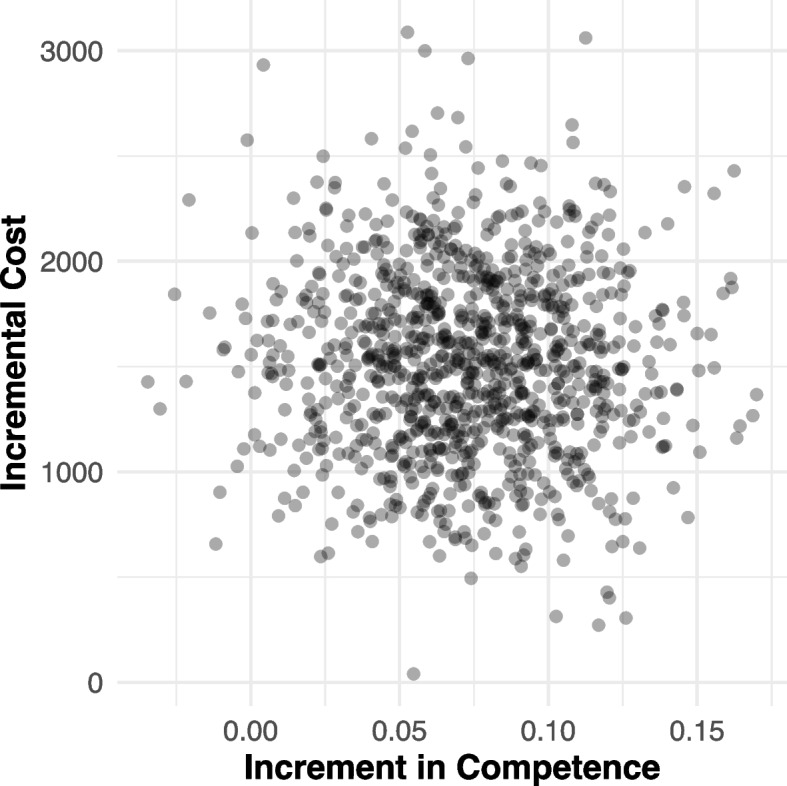
Scatterplot of of incremental costs and adherence outcomes on the cost-effectiveness plane for the TTT implementation strategy generated by bootstrapped re-sampling

Figure 1 displays the uncertainty in the relationship between the incremental costs of training (i.e., a mean of $1,352 more to train a therapist in the TTT condition), and the incremental improvement in competence (0.073 higher for therapists in the TTT condition). Of 1000 replications, there are only 18 instances of a point falling to the left of 0 on the X axis, indicating that higher costs produced worse outcomes. The overwhelming majority of instances fall to the right of 0, indicating that this increased cost produced gains in competence. The large clustering of points between $500 and $2,500 on the incremental cost axis, and between 0.025 and 0.125 on the incremental gain in competence axis, suggests that counseling centers can reasonably expect to receive positive returns on their increased investments in the TTT condition.

### Results of sensitivity analyses

(i) Eight out of 12 centers in the TTT condition trained not only therapists participating in the study (“study therapists”), but other clinicians at their centers who were not part of the study (“non-study therapists”). The total number of non-study therapists trained was 46. Each center that trained non-study therapists experienced cost savings on a per-therapist basis that ranged between $910 (for a center that trained 6 study therapists and 19 non-study therapists), and $60 (for a center that trained 6 study therapists and one additional non-study therapist). If non-study therapists achieved gains in competence equivalent to those of study therapists, then the mean investments required to produce 1 unit gain in competence would fall to $17,084 if non-study therapists are included. The cost savings over the expert consultation condition would further widen from $353 to $2,302 for a counseling center.
(ii) Differences in training intensity for trainers had significant effects upon per-therapist costs. At the 10th percentile of training time for trainers, mean costs per therapist were $3,009 (median: $2,798), and at the 90th percentile of training time, mean costs per therapist were $3,986 (median: $3,775). A counseling center would then spend $16,810 in training if all of their trainers were highly skilled, and $22,268 for trainers requiring more training, assuming that these training variations resulted in identical therapist fidelity outcomes.
(iii) In the expert consultation condition, per therapist training costs averaged between $1,251 and $2,646 (mean: $2,026) for therapists who had baseline adherence scores above the mean, and between $965 and $5,288 (mean: $2,064) for the remaining low-adherence therapists. Per-therapist training costs ranged between $890 and $3,149 (mean: $2,077) for therapists who had baseline competence scores above the mean, and between $965 and $5,288 (mean: $2,048) for the remaining therapists. In the TTT condition, per-therapist costs for therapists who had baseline adherence scores above the mean ranged between $1,943 and $7,073 (mean: $3,787), and between $1,892 and $7,162 (mean: $3,240) for the remaining therapists. Per-therapist training costs for those therapists whose baseline competence scores were above the mean ranged between $1,892 and $4,822 (mean: $3,467). Training costs for the rest of the therapists in this condition, whose baseline competence scores were below the mean, ranged between $1,944 and $7,161 (mean: $3,386).
(iv) In the expert consultation condition, therapists reported spending between 1 and 40 h (mean: 10.4 h) in training and consultation with the expert while the expert’s tracking logs indicated a range between 0 and 9 h per therapist (mean: 4.3 h). Mean per-therapist training costs using therapists’ self-reported time ($2,055) was higher than costs using the expert’s tracking log ($1,784) by around 15%.
(v) Non-missing data revealed that the 10th percentile of therapist time taken for preparation activities before the site workshop was 2 h, and for activities after the workshop was 2.1 h. The respective 90th percentile figures were 5 and 8.9 h. Using these values for lower and upper bounds, mean per-therapist training costs in the TTT condition ranged between $3,395 and $3,478.

## Discussion

In this study, we found significant differences between the TTT and expert consultation implementation strategies in the costs involved in training therapists to deliver IPT within college counseling centers. Mean costs incurred by counseling centers to train one therapist using the TTT training model in this study amounted to $3,407 (median = $3,077); mean cost to produce one trained therapist in the expert consultation condition was $2,055 (median = $1,932). The range of training costs to produce one therapist using the TTT strategy varied between $2,148 and $7,117, while those to produce one therapist using the expert consultation strategy ranged between $1,335 and $3,661. Hence, the cost to produce a therapist using TTT was not always higher for all centers, suggesting a need for sites to carefully conduct their own cost estimates, and determine if investments in one training strategy versus another make sense given their own cost structures.

These investments produced improvements in therapist competence, but not in adherence. Counseling centers that used the TTT model incurred $353 less in training costs to produce equivalent improvements in therapist competence. There is not yet a body of work in IPT that shows the effects of gains in therapist competence on improved clinical outcomes in the way that there is for several other interventions, such as Multisystemic Therapy [21], Cognitive Processing Therapy [22, 23], cognitive therapy [24], and Parent Management Training – Oregon model [25]. However, it seems reasonable to assume that the relationship between increases in therapist fidelity scores and improved clinical outcomes will also apply to IPT, in the same way as it applies to these other psychotherapy interventions.

These short run costs are conservative. The expert consultation model in this study was intensive, contributing to heightened costs, and downwardly biasing differences in costs between it and the TTT condition. Setting aside the costs of training the trainer, and including only study therapists, each therapist costs $234 less ($1,821 for TTT versus $2,055) to train in the TTT condition than in the expert consultation condition, largely because there are no ongoing costs that have to be paid to the outside trainer. If a counseling center can procure a grant, or access funds to train a trainer, a not uncommon phenomenon in these settings [26], cost savings can accrue to the center with the training of the very first therapist.

While the study demonstrated statistically significant improvements in competence, improvements in adherence were not significant. This did not seem to be because some of the participating therapists were familiar with IPT even before the study began [2]; as described in the sensitivity analyses for pre-training variations in therapist adherence and competence, the effects upon training costs of these variations appear to be modest. Alternatively, perhaps because therapists were aware they were being recorded as they conducted their sessions, they may have exhibited research participation (Hawthorne) effects [27], further downwardly biasing differences between conditions. While a discussion of the therapist- and intervention-level factors that underlie the adherence vs competence findings are beyond the scope of this study, competence ratings are measures of the quality of service delivery. As such, this evaluation makes the case for investments in TTT approaches as a quality improvement strategy. Our research has also found that the TTT approach, as compared to expert consultation, was associated with significantly greater maintenance of training effects (for both adherence and competence) over time [28]. These findings provide additional support for the idea of investing in TTT for quality improvement, which will likely result in cost savings over time, and warrants further study.

As our sensitivity analysis reveals, increasing the numbers of therapists trained produces economies of scale because fixed costs of the trainer can be spread across many trainees. At the 8 sites that trained non-study therapists, variable costs for both study as well as non-study therapists (average of $894 per therapist) were lower than the $1,821 amount per study therapist incurred by all TTT sites. Enterprising centers can even train therapists unaffiliated with their counseling center, developing a business model for implementation.

On the supply side, expending a mean of $8,194 to develop a trainer represents a moat for counseling centers in the training marketplace. Having a skilled clinician who is, ideally, already expert in delivering IPT, reduces costs of training and, consequently, per-therapist costs.

These cost implications can be affected by turnover. Around 61% of college and university counseling centers experience turnover of at least one member of their staff each year [29]. Centers can mitigate trainer turnover risk by using their trainer to proactively train additional in-house trainers. Training can also be shifted from an individual to a digital platform, as has been demonstrated for family-based behavioral treatment [30], and which has been initially demonstrated for IPT as well [31]. Of course, experts can also leave a training situation, in which case the center needs to find an alternative outside expert to complete the training regimen. The underlying reasons for staff turnover in counseling centers are varied [29], and addressing them is likely to have significant cost implications. Assuring retention of the trainer is key to the cost-effectiveness of the TTT implementation strategy.

These findings have to be tempered by a few characteristics and limitations of this study. First, we cannot validate self-reported times for therapists trained in the TTT condition. As our sensitivity analysis for the expert consultation condition revealed, therapists’ self-reported times resulted in an approximately 15% increase in mean per-therapist training costs. We do not know if this 15% increase also applies to training costs in the TTT condition. If therapists in the expert condition (but not those in the TTT condition) are predisposed to overestimate their time, then this increases costs of the expert consultation condition, produces a smaller difference in costs between the two conditions, and hence is a more conservative measure of the economic impact of the TTT strategy. Of course, the reverse is also possible, when therapists in the expert condition under-report their time. Given the cluster randomization, this is unlikely, and hence we present self-reported estimates across both conditions. Second, the dominant drivers of implementation costs in these settings are provider salaries, which varied multifold in our study. The counseling center-level randomization guards against the critique that these estimates are an underestimate of costs for, say, coastal private colleges, while simultaneously being an overestimate of costs for Midwestern public institutions. Third, we recognize that using mean substitution approaches to fill in missing data values are problematic [32]. However, all participants in a group can be reasonably expected to have identical values for time spent in the same training group at the same time in the same center. Sensitivity analysis of time spent by therapists in the individual activities (time spent in reading materials before and after training), as described earlier, revealed a relatively narrow range between $3,395 and $3,478 for mean therapist training costs in the TTT condition. Finally, accounting studies inevitably involve questions of value – is a saving of $353 sufficient for a counseling center director to choose to implement an EBT using a TTT approach, given its added complexity and many moving parts? While this is best answered by the center director, our study demonstrates the fiscal viability of the TTT implementation strategy for the delivery of behavioral health interventions in counseling centers and supports the expansion of resources to support this strategy.

## Conclusion

This study demonstrates the cost-effectiveness of the train-the-trainer (TTT) implementation strategy for the delivery of IPT. The TTT implementation strategy produces higher short run costs but greater increases in therapist competence. Supporting TTT models can be an effective way to enhance the mental health care of young people seeking care in college and university counseling centers.

### Supplementary Information

Supplementary Material 1.

## Data Availability

The datasets generated and/or analyzed during the current study are not publicly available due to participant privacy concerns, but are available from the corresponding author on reasonable request.

## Abbreviations

EBT: Evidence-based treatment
IPT: Interpersonal psychotherapy
TTT: Train-the-trainer
