# Supplementary material for: Cost-effectiveness of train-the-trainer versus expert consultation training models for implementing interpersonal psychotherapy in college mental health settings: evidence from a national cluster randomized trial
Source: Implement Sci. 2024 Jul 29;19:55. doi: 10.1186/s13012-024-01388-2 (PMC11287912; doi:10.1186/s13012-024-01388-2)
Supplement: Supplementary file 1 — Supplementary Material 1. [file 13012_2024_1388_MOESM1_ESM.docx]

**Supplement 1: Implementation Cost Chart for the Train the Trainer Condition**

**Site:**

|  | Time in hours (a) | Hourly wage (inclusive of fringe benefits) (b) | Cost (a)*(b) = (c.) |
| --- | --- | --- | --- |
| 1. **Labor Costs** |  |  |  |
| **Prework TTT Workshop 1** |  |  |  |
| *Expert* |  |  |  |
| Preparing Training Materials |  |  |  |
| *Research Staff* |  |  |  |
| Planning Training |  |  |  |
| Preparing training materials |  |  |  |
| *Trainers attending workshop* |  |  |  |
| Reading pre-training materials |  |  |  |
| Time traveling to workshop |  |  |  |
| **Training – TTT Workshop 1** |  |  |  |
| *Expert* |  |  |  |
| Conducting Training |  |  |  |
| *Research staff/ Training support* |  |  |  |
| Attend training |  |  |  |
| *Trainers attending workshop* |  |  |  |
| Time cost of participation |  |  |  |
| **Prework – TTT Workshop 2** |  |  |  |
| *Expert* |  |  |  |
| Preparing Training Materials |  |  |  |
| *Research Staff* |  |  |  |
| Planning Training |  |  |  |
| Preparing training materials |  |  |  |
| *Trainers attending workshop* |  |  |  |
| Reading pre-training materials |  |  |  |
| Time traveling to workshop |  |  |  |
| **Training – TTT Workshop 2** |  |  |  |
| *Expert* |  |  |  |
| Conducting Training |  |  |  |
| *Research staff/ Training support* |  |  |  |
| Attend training |  |  |  |
| *Trainers attending workshop* |  |  |  |
| Time cost of participation |  |  |  |
| **Consultation** |  |  |  |
| *Expert* |  |  |  |
| Time conducting consultation |  |  |  |
| Time listening to recordings for consultation |  |  |  |
| *Trainers* |  |  |  |
| Time cost of participation in consultation |  |  |  |
| **PreTraining at PCC** |  |  |  |
| *Trainers* |  |  |  |
| Preparing for training |  |  |  |
| *Participating Therapists* |  |  |  |
| Reading pre-training materials |  |  |  |
| *Research Staff* |  |  |  |
| Time preparing materials for training |  |  |  |
| *Admin at PCC* |  |  |  |
| Time planning logistics of training at site |  |  |  |
| **Training at PCC** |  |  |  |
| *Admin at PCC* |  |  |  |
| Time spent during training that required costs? |  |  |  |
| *Trainers* |  |  |  |
| Conducting training |  |  |  |
| *Participating Therapists* |  |  |  |
| Attending Training |  |  |  |
| **Post Training** |  |  |  |
| *Trainers* |  |  |  |
| Conducting supervision |  |  |  |
| Listening to recordings of therapists |  |  |  |
| Consultation with expert |  |  |  |
| *Participating Therapists* |  |  |  |
| Attending Supervision |  |  |  |
| Reviewing training materials |  |  |  |
| *Expert* |  |  |  |
| Consultation with trainers |  |  |  |

|  | Units (a) | Cost per unit (b) | Cost (a)*(b) = (c.) |
| --- | --- | --- | --- |
| 1. **Nonlabor Costs** |  |  |  |
| **TTT Training Workshop 1:** |  |  |  |
| Training location room cost |  |  |  |
| Cost of curriculum (manual and materials) |  |  |  |
| Cost of recording training |  |  |  |
| Cost of creating DVDs |  |  |  |
| Shipping of DVDs |  |  |  |
| Airfare |  |  |  |
| Hotel stay |  |  |  |
| Meals and other expenses |  |  |  |
| Mileage/Gas |  |  |  |
| Ground transportation |  |  |  |
| **TTT Training Workshop 2:** |  |  |  |
| Training location room cost |  |  |  |
| Cost of curriculum (manual and materials) |  |  |  |
| Cost of recording training |  |  |  |
| Cost of creating DVDs/DVDs (if applicable) |  |  |  |
| Shipping of DVDs (if applicable) |  |  |  |
| Airfare |  |  |  |
| Hotel stay |  |  |  |
| Meals and other expenses |  |  |  |
| Mileage/Gas |  |  |  |
| Ground transportation |  |  |  |
| **Consultation** |  |  |  |
| Telephone charges (pre and post TTT#2 workshop) |  |  |  |
| **Training at PCC** |  |  |  |
| Costs of materials |  |  |  |
| Shipping materials |  |  |  |
| Cost of location of training | (sq. ft) | Zip code: |  |
| Cost of meals during workshop |  |  |  |
| **Miscellaneous** |  |  |  |
| Recorders |  |  |  |
| Shipping Recorders |  |  |  |

PCC = Participating counseling center

TTT = Train-the-trainer
